# Causal link between oxidative stress and epilepsy: A two‐sample Mendelian randomization study

**DOI:** 10.1002/brb3.3549

**Published:** 2024-06-07

**Authors:** Yilin Xia, Wanlin Lai, Leihao Sha, Yifei Duan, Lei Chen

**Affiliations:** ^1^ Department of Neurology, West China Hospital Sichuan University Chengdu China; ^2^ Pazhou Lab Guangzhou China

**Keywords:** epilepsy, Mendelian randomization study, oxidative stress

## Abstract

**Background:**

Although a growing body of research has indicated a strong link between oxidative stress and epilepsy, the exact nature of their interaction remains elusive. To elucidate this intricate relationship, we conducted a bidirectional Mendelian randomization (MR) analysis employing two independent datasets.

**Methods:**

A two‐sample MR analysis was performed using instrumental variables derived from genome‐wide association study summary statistics of oxidative stress injury biomarkers (OSIB) and epilepsy. The OSIBs were selected from eight primary metabolic pathways associated with oxidative stress. Additionally, seven distinct epilepsy phenotypes were considered, which encompassed all epilepsy, generalized epilepsy, generalized tonic‐clonic seizures, focal epilepsy, focal epilepsy with hippocampal sclerosis (focal HS), focal epilepsy with lesions other than HS (focal NHS), and lesion‐negative focal epilepsy. Causal estimates were computed using the inverse‐variance weighted method or the Wald ratio method, and the robustness of causality was assessed through sensitivity analyses.

**Results:**

For OSIB and epilepsy, 520 and 23 genetic variants, respectively, were selectively extracted as instrumental variants. Genetically predicted higher kynurenine level was associated with a decreased risk of focal epilepsy (odds ratio [OR] 1.950, 95% CI 1.373–2.528, *p* = .023) and focal NHS (OR 1.276, 95% CI 1.100–1.453, *p* = .006). For reverse analysis, there was a suggestive effect of focal NHS on urate (OR 1.19 × 10^15^, 95% CI 11.19 × 10^15^ to 1.19 × 10^15^, *p* = .0000746) and total bilirubin (Tb) (OR 4.98, 95% CI 3.423–6.543, *p* = .044). In addition, genetic predisposition to focal HS was associated with higher Tb levels (OR 9.83, 95% CI 7.77–11.888, *p* = .034).

**Conclusion:**

This MR study provides compelling evidence of a robust association between oxidative stress and epilepsy, with a notable emphasis on a causal relationship between oxidative stress and focal epilepsy. Additional research is warranted to confirm the connection between oxidative stress and the risk of epilepsy and to unravel the underlying mechanisms.

## Graphical abstract

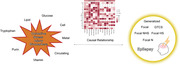

## 1 INTRODUCTION

Epilepsy is a chronic and intricate neurological disorder characterized by sudden and abnormal electrical discharges in the brain (de Boer et al., 2008; Thijs et al., 2019). Epilepsy is estimated to impact more than 70 million individuals worldwide, resulting in substantial financial implications due to healthcare requirements, premature mortality, and reduced workplace productivity (de Boer et al., 2008; Thijs et al., 2019). Epilepsy displays a considerable degree of heterogeneity, with both generalized epilepsy and focal epilepsy falling under its spectrum. Generalized epilepsy is typically diagnosed when generalized spike‐wave activity is observed on electroencephalography, whereas focal epilepsy pertains to unifocal or multifocal disorders characterized by seizures originating in a specific hemisphere (de Boer et al., 2008). Moreover, contributing factors to the etiology of epilepsy encompass aspects like hippocampal sclerosis (HS), hereditary influences, cerebrovascular diseases, traumatic brain injuries, and brain infections (Shorvon, 2011).

In recent years, the role of oxidative stress in the pathophysiology of epilepsy has begun to receive widespread attention (Olowe et al., 2020). This focus has been supported by studies involving both epilepsy patients and animal models, which have identified a robust bidirectional relationship by observing changes in biomarker levels associated with oxidative stress‐induced damage in various pathways (Evans et al., 2013; Shin et al., 2014). Specifically, these investigations have revealed that the excessive production of reactive oxygen species during seizures can lead to the disruption of multiple metabolic pathways, resulting in effects such as lipid peroxidation, DNA damage, enzyme inhibition, and mitochondrial impairment (Sun et al., 2018). However, it is important to note that there is currently insufficient evidence to establish causality definitively, primarily due to the influence of confounding factors such as the use of antiepileptic drugs, variations in seizure patterns, and differences in interictal periods (Lawlor et al., 2008).

Mendelian randomization (MR) is a newly developed analytical method that has found extensive use in inferring the causal effects of exposures on outcomes (Burgess et al., 2013). Especially in cases where randomized controlled trials are either unavailable or challenging to initiate, MR has emerged as a pivotal strategy to furnish dependable evidence regarding the causal connections between exposures and disease risk (Bowden et al., 2015). In this study, a two‐sample bidirectional MR investigation was undertaken to elucidate the causal relationship between oxidative stress and epilepsy.

## 2 MATERIALS AND METHODS

### 2.1 Study design

**FIGURE 1:**
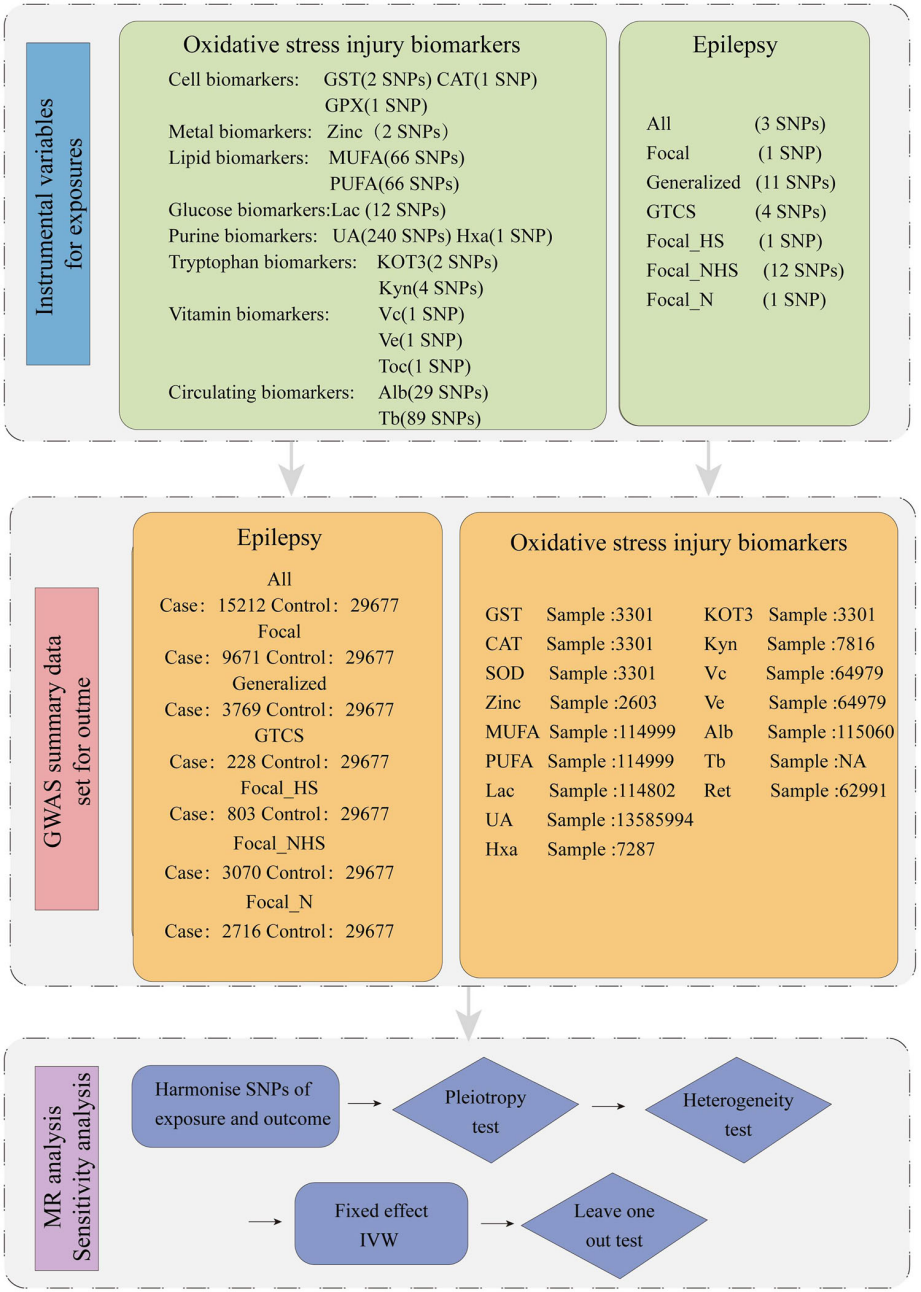
Study design.

This study is reported according to the STROBE‐MR guideline (Skrivankova et al., 2021). The study design's flowchart is depicted in Figure 1. We performed a two‐sample MR bidirectional study using single‐nucleotide polymorphisms (SNPs) to assess the causal association of seven oxidative stress pathways with seven epilepsy phenotypes. All the genome‐wide association study (GWAS) cohorts in our study were European and had no overlap with each other. MR relies on the following three assumptions: First, genetic instruments of the exposure of interest are strongly associated with exposure; second, genetic instruments of exposure are not correlated with exposure‐outcome association confounders; and third, genetic instruments affect the outcome only by affecting the exposure of interest (Kim et al., 2023). All the available GWAS summary statistics data were gained from the Medical Research Council Integrative Epidemiology Unit OpenGWAS project (https://gwas.mrcieu.ac.uk/), and GWAS Catalog (https://www.ebi.ac.uk/gwas/) with relevant participant consent and ethically approved; therefore, no ethical approval from an ethics committee was required.

### 2.2 Data source for the genetic instruments for oxidative stress

We obtained summary data from published GWAS studies evaluating European ancestry for oxidative stress injury biomarkers (OSIBs) of cell metabolism (glutathione *s*‐transferase [GST], catalase, and glutathione peroxidase) (Sun et al., 2018), metal metabolism (zinc; Evans et al., 2013), lipid metabolism (polyunsaturated fatty acids [PUFA] and monounsaturated fatty acids [MUFA]), glucose metabolism (lactate [LAC]), purine metabolism (uric acid, and hypoxanthine (Shin et al., 2014)), tryptophan metabolism (kynurenine–oxoglutarate transaminase 3 (Sun et al., 2018) and kynurenine [Kyn] (Shin et al., 2014)), vitamin (vitamin C [Vc], vitamin E [Ve], and tocopherol (Shin et al., 2014)), and circulate metabolism (albumin [Alb] and total bilirubin [Tb]). For genetic instruments of oxidative stress, we used independent SNPs (linkage disequilibrium [LD] *r* ^2^ < .001, >10,000 kb) that were strongly associated with each exposure (*p* < 5 × 10^−8^) as instruments (shown in Tables S1 and S2D2).

### 2.3 Data on the genetic predicted risk of epilepsy

We obtained relevant GWAS summary data for seven distinct epilepsy phenotypes from the International League Against Epilepsy (ILAE) Consortium on Complex Epilepsies in 2018. These phenotypes encompassed a broad spectrum of epilepsy conditions, including all epilepsy, generalized epilepsy, generalized tonic‐clonic seizures (GTCS), focal epilepsy, focal epilepsy with HS (focal HS), focal epilepsy with lesions other than HS (focal NHS), and lesion‐negative focal epilepsy (Jackson et al., 2016). The classification of cases within these categories was performed by a specialized epilepsy expert, taking into account electroencephalogram results, imaging studies, and clinical histories. For all epilepsy, generalized epilepsy and focal epilepsy, the selection of SNPs was based on their association with the exposure factor, reaching genome‐wide significance (*p* value < 5 × 10^−8^) in publicly available GWASs, while ensuring that these SNPs were not in linkage disequilibrium (LD) with other SNPs (*r* ^2^ < .01) within a clumping window of 10,000 kb. For exposures such as GTCS, focal HS, focal NHS, and focal N, where no available instruments were meeting the criteria, the significance threshold for the selection of SNPs was adjusted to 5 × 10^−6^.

### 2.4 Statistical analysis

Wald ratio calculations were performed for each SNP, offering the SNP‐outcome association divided by the SNP‐exposure association (Lawlor et al., 2008). Inverse‐variance weighted (IVW), MR‐Egger, single mode, weighted‐median, and weighted‐mode analysis were used to evaluate the effect when multiple SNPs were available (Bowden et al., 2015; Burgess et al., 2013). *p* < .05 was considered suggestive evidence for a potential causal association.

For sensitivity analyses, we checked whether the genetic instruments were associated with other phenotypes using PhenoScanner V2 in the case of the potential association between SNPs and confounding factors (Kamat et al., 2019). None of the instrumental SNPs showed a direct association with outcome. We also applied sensitivity analysis for IVW, MR‐Egger, weighted‐median, weighted‐mode, and MR pleiotropy residual sum and outlier (MR‐PRESSO) to test the reliability and stability of the main MR assumptions. Meanwhile, we used scatter plots and Cochran's *Q*‐test to check the heterogeneity. Leave‐one‐out analyses were performed to examine whether a single SNP is driving the causal association.

All statistical analyses were carried out using the two‐sample MR package (version 0.5.7) and MR‐PRESSO (version 1.0) within the R 4.2.1 environment (the R Foundation for Statistical Computing).

## 3 RESULTS

Overall, GWASs of 17 OSIB and 7 epilepsy phenotypes were analyzed in the MR analysis (Figure 1). A total of 520 SNPs were selectively extracted to proxy OSIBs, and 23 genetic variants were chosen for epilepsy. The *F*‐statistics for all instrumental variables used in our study were >10. Detailed information on instrumental variations for each exposure factor is shown in Table S3.

### 3.1 MR results of oxidative stress on the risk of epilepsy

**FIGURE 2:**
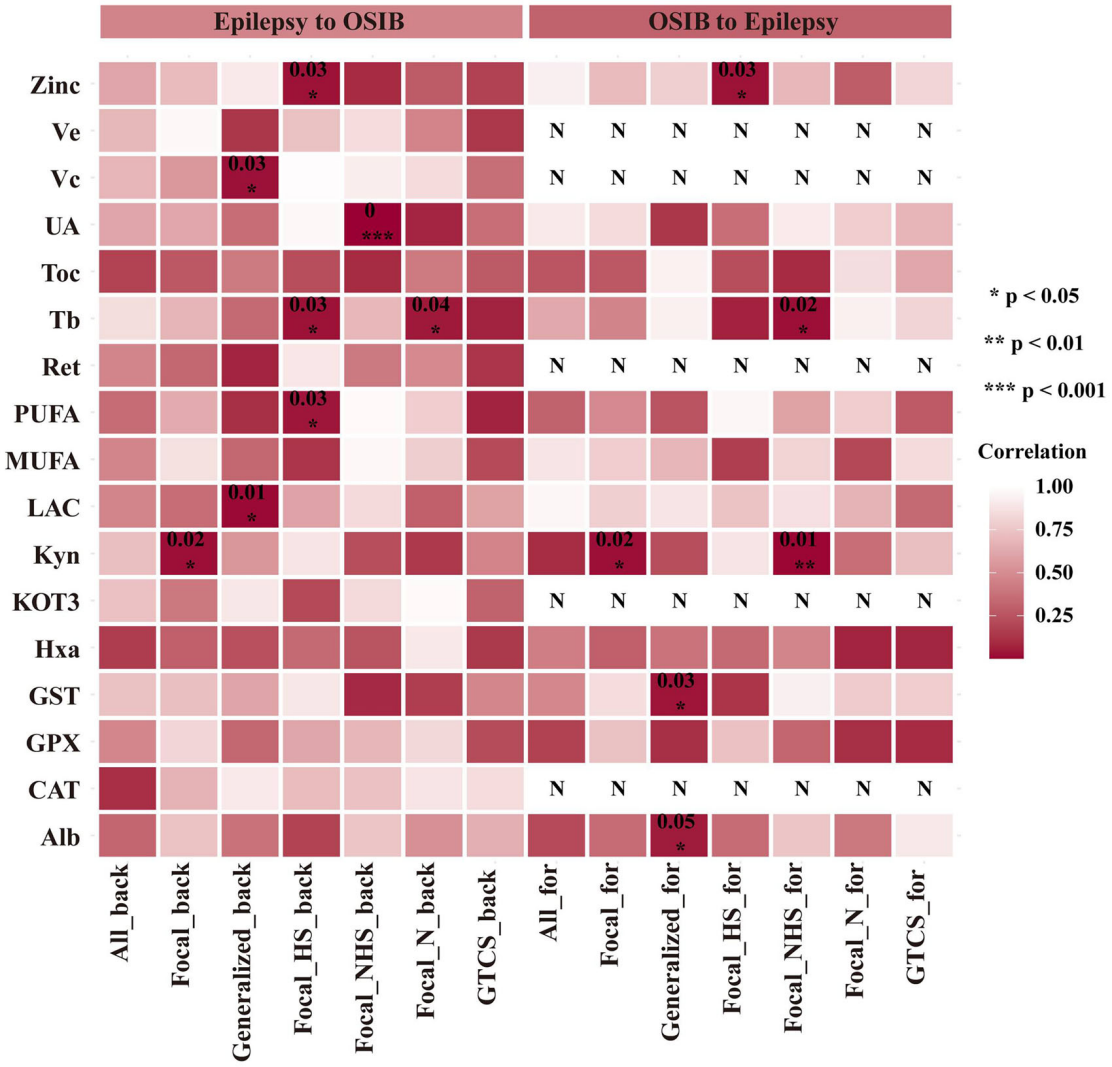
Causality of oxidative stress injury biomarkers and epilepsy by inverse‐variance‐weighted (IVW) method.

**FIGURE 3:**
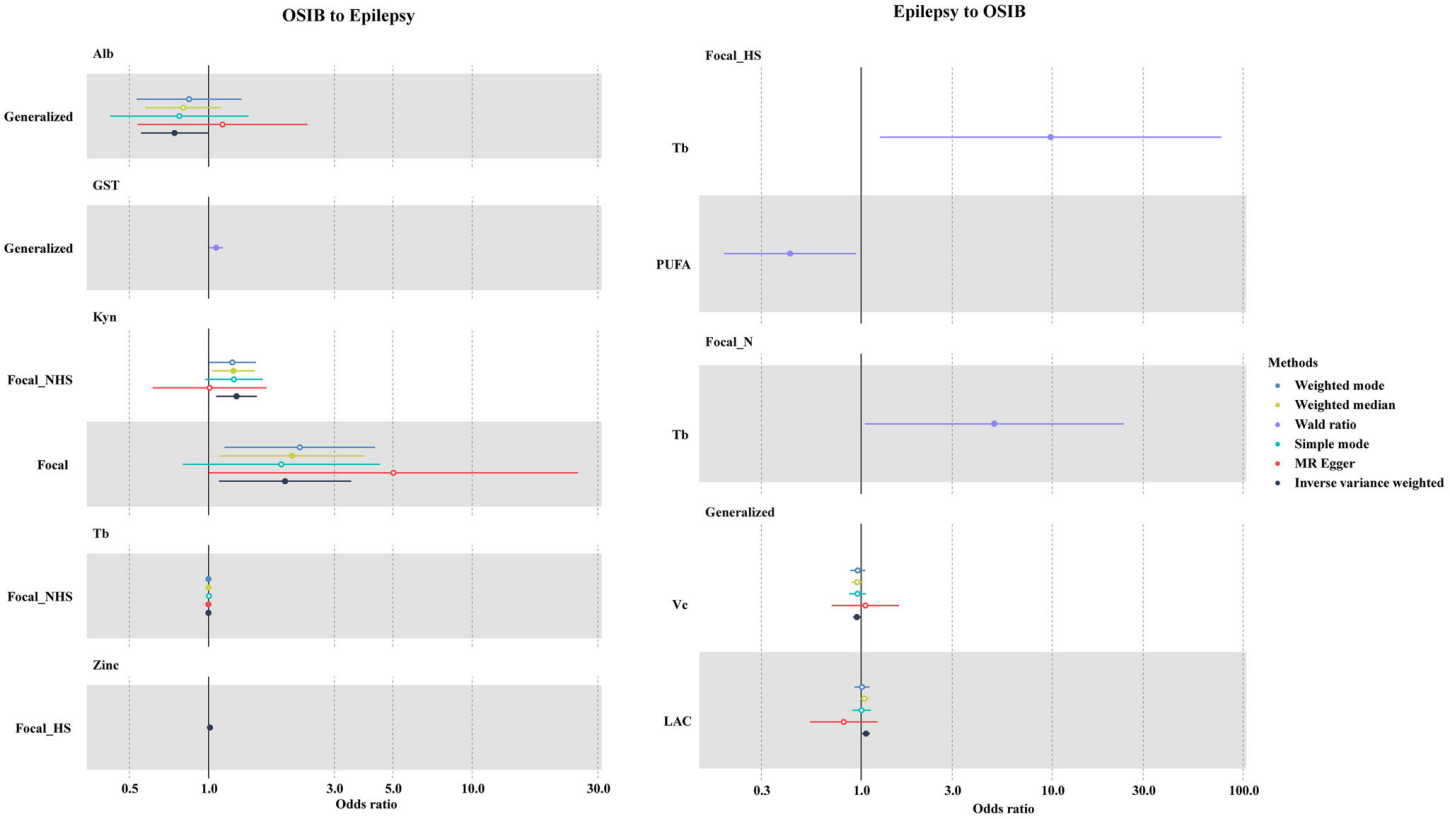
Causal relationships between oxidative stress injury biomarkers and epilepsy by inverse‐variance weighted (IVW) method, Mendelian randomization (MR)‐Egger, weighted‐median, weighted‐mode, and MR pleiotropy residual sum and outlier (MR‐PRESSO).

The results from the IVW models indicate nominal positive correlations between genetic predispositions to multiple OSIBs and the risk of focal epilepsy, generalized epilepsy, focal epilepsy with HS, and focal epilepsy with lesions other than HS (as illustrated in Figure 2). As shown in Figure 3, elevated Kyn levels are noteworthy factors that predispose individuals to a reduced risk of focal epilepsy (odds ratio [OR] 1.950, 95% CI 1.373–2.528, *p* = .023) and focal NHS (OR 1.276, 95% CI 1.100–1.453, *p* = .006). The risk of generalized epilepsy is also notably influenced by Alb levels (OR.742, 95% CI.450–1.035, *p* = .046) and GST levels (OR 1.067, 95% CI 1.007–1.127, *p* = .032), with Alb associated with a lower risk and GST associated with a higher risk. There was a potential causal association between Tb level and focal epilepsy with lesion other than hippocampus sclerosis (OR.998, 95% CI.997–1.000, *p* = .021).

**FIGURE 4:**
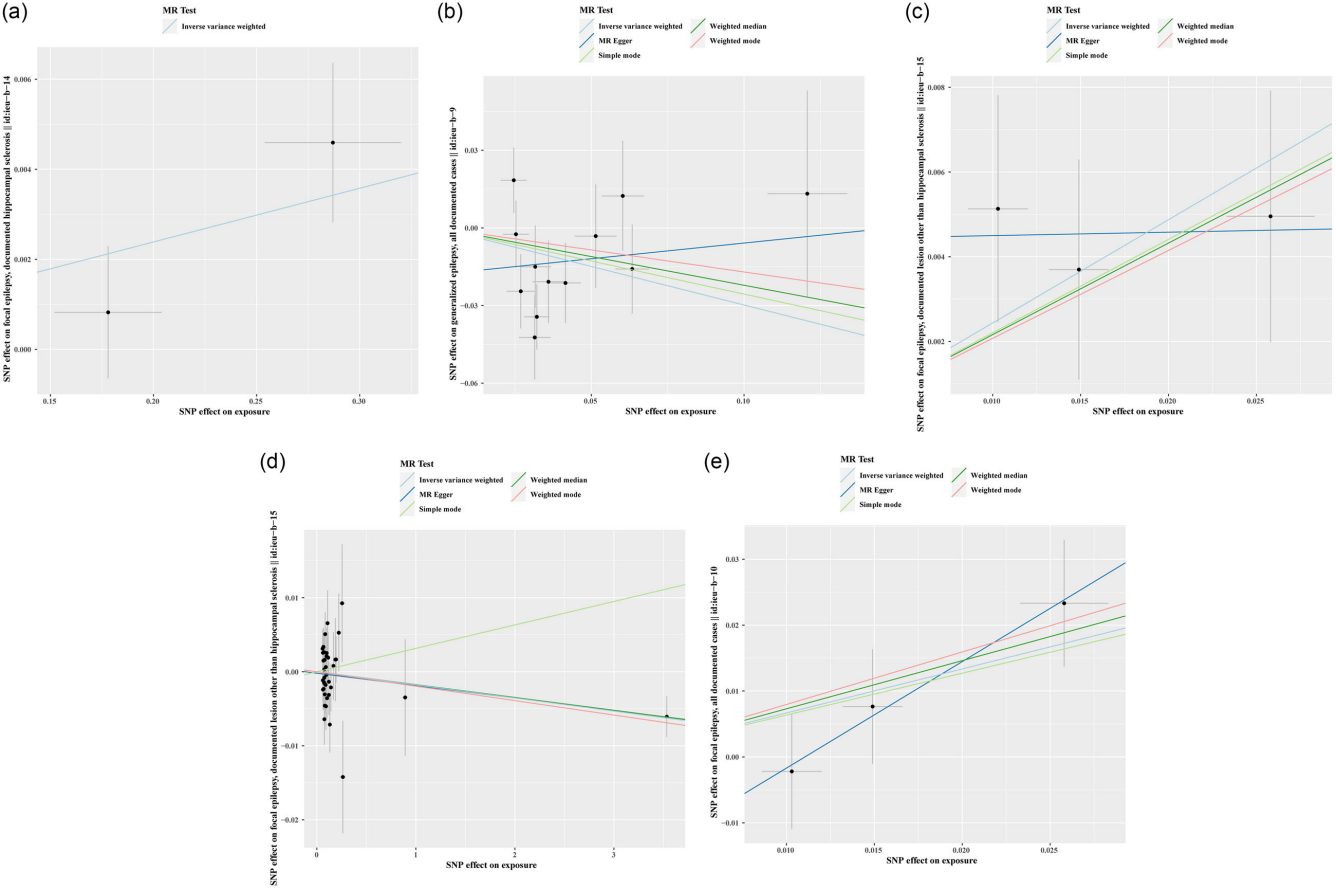
The scatterplots represent genetic inverse variances (IVs) association between epilepsy and oxidative stress injury biomarkers (a) Zinc on Focal_HS, (b)Alb on Generalized, (c)Kyn on Focal_NHS, (d)Tb on Focal_NHS, (e) Kyn on Focal

Additionally, the level of zinc increases the genetic susceptibility of focal epilepsy with hippocampus sclerosis (OR 1.012, 95% CI 1.001–1.023, *p* = .029). However, the potential link between OSIBs and epilepsy was no longer evident when using the MR‐Egger method, weighted‐median method, simple‐mode method, and weighted‐mode method, except in the case of Tb and focal NHS. The significance and magnitude of all these associations were consistent (OR = .998, 95% CI.997–1.000; *p* = .048 for MR‐Egger; OR = .998, 95% CI.997–1.000; *p* = .020 for weighted‐median; OR = .998, 95% CI.997–1.000; *p* = .023 for weighted‐mode). No significant associations are observed when it comes to all epilepsy, GTCS, and lesion‐negative focal epilepsy. Detailed MR results of the OSIBs on the risk of epilepsy with scatter plots are shown in Figure 4 and Table S4.

### 3.2 MR results of epilepsy on oxidative stress

Reverse directional MR analysis reveals a significant causal relationship between specific epilepsy phenotype and OSIBs (Figure 2). In the IVW MR analyses, the following key findings were observed: (1) Focal epilepsy with lesion other than hippocampus sclerosis significantly increases the level of urate (OR 1.19 × 10^15^, 95% CI 11.19 × 10^15^ to 1.19 × 10^15^, *p* = .0000746). (2) There is a potential causal effect of focal epilepsy with HS on the levels of increased Tb (OR = 9.830, 95% CI 7.773–11.888, *p* = .029) and PUFA (OR = .424, 95% CI.370–1.219, *p* = .035) was observed. (3) Lesion‐negative focal epilepsy was identified as a predisposing contributor for increasing Tb level (OR = 4.983, 95% CI 3.423–6.543,*p* = .044). (4) A positive association was observed between generalized epilepsy and Vc levels (OR = .950, 95% CI.905–.995, *p* = .025) and LAC levels (OR = 1.060, 95% CI 1.013–1.106, *p* = .014).

**FIGURE 5:**
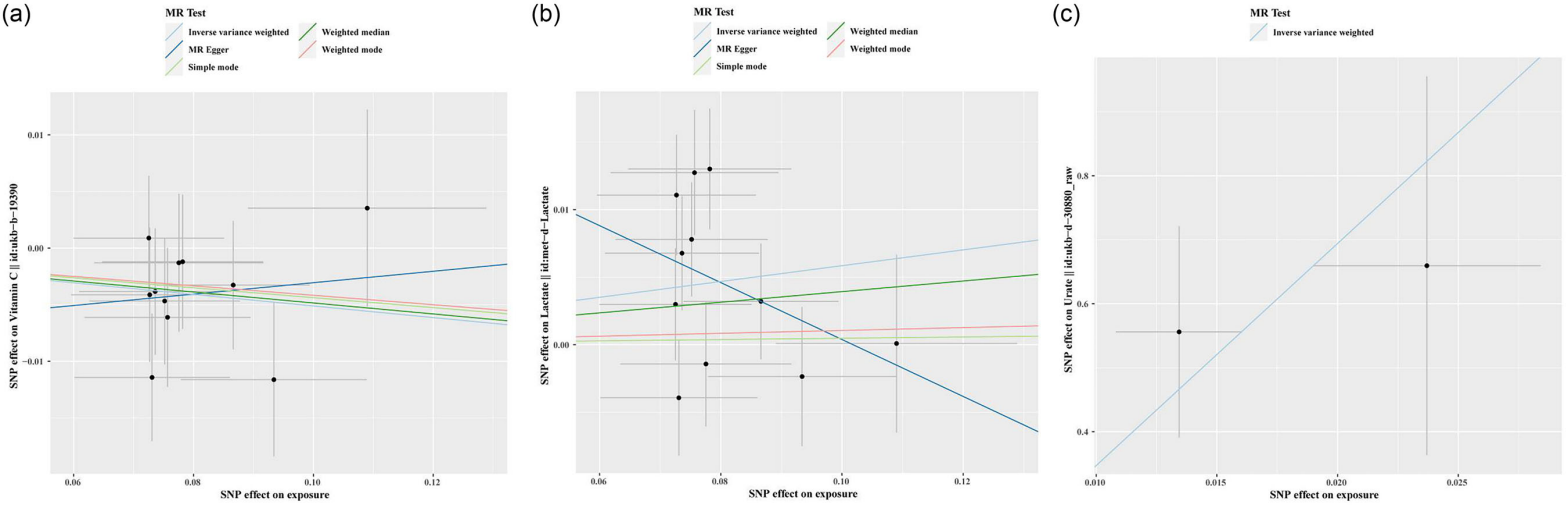
The scatterplots represent genetic inverse variances (IVs) association between oxidative stress injury biomarkers and epilepsy. (a)Generalized on Vc, (b) Generaralized on LAC, (c)Focal_HS on Urate

However, the associations between generalized epilepsy and OSIBs were not observed in other analytical methods. There was potential evidence of heterogeneity, and the causal estimates between generalized epilepsy and LAC were imprecise (Tables S5 and S6). Detailed MR results of risk of epilepsy on the OSIBs with scatter plots are shown in Figure 5 and Table S7.

### 3.3 Sensitivity analyses

Leave‐one‐out analyses proved that the causal estimates were not driven by any single SNP (Tables S8 and S9). No evidence of pleiotropy was found using the MR‐Egger intercept test or the MR‐PRESSO test among potential causal estimations (Tables S6, S10, S11 and S12 ). The sensitivity analyses did not reveal any indication of significant evidence of heterogeneity in the SNPs of OSIBs among positive MR results (Table S13). In addition, the *F*‐statistics for the IVs ranged from 25.31 to 142,043.12, suggesting that our MR study did not have weak instrument bias.

## 4 DISCUSSION

This study investigated the causal associations between 7 epilepsy phenotypes and 17 OSIB using large‐scale summary data obtained from the ILAE and other GWAS studies within an MR framework. The current work demonstrated that genetically predicted focal NHS was significantly associated with increased levels of urate. Furthermore, we provided tentative evidence suggesting a potential causal effect of focal epilepsy with HS and lesion‐negative focal epilepsy on Tb levels, focal epilepsy with HS on PUFA, and generalized epilepsy on Vc and LAC levels. No significant associations could be found in the reverse direction. Nevertheless, we found that several associations suggested a possible causal effect of Kyn level on focal epilepsy and focal NHS, GST, and Alb level on generalized epilepsy, Tb level on focal NHS, zinc level, and focal HS. To our knowledge, this is the first MR study to evaluate the bidirectional causal association between epilepsy and oxidative stress.

Our finding of a causal association between the focal NHS and urate is partly consistent with two cross‐sectional population‐based studies, which compared untreated epilepsy patients to healthy controls (Aycicek & Iscan, 2007; Hamed et al., 2004). Epileptic foci other than HS may result from a variety of causes such as stroke, trauma, tumors, and developmental malformations (Nascimento et al., 2023). The level of serum urate levels has been reported to be elevated in epilepsy secondary to cerebral infarction and trauma (Jackson et al., 2016; Wang et al., 2019). As the end product of purine metabolism and a crucial natural antioxidant of the human body, the urate level may reflect the upregulation of oxidative stress in secondary epilepsy after brain injury (Ait Tayeb et al., 2023; Oses et al., 2007). Nonetheless, it is essential to regard this association as a serendipitous discovery that warrants validation in future research endeavors. This study also revealed that generalized epilepsy has causal effects on Vc and LAC, as documented in past observational studies (Das et al., 2019; Magnusson et al., 2021; Nass et al., 2019). Elevation of LAC in generalized seizures has been widely used in the differential diagnosis of psychogenic nonepileptic seizures, whereas decreased levels of Vc require further validation (Olaciregui Dague et al., 2018; Patel et al., 2022). Additionally, our study has revealed a causal link between focal epilepsy accompanied by HS and PUFA. Previous research has shown that the ketogenic diet, which is used to treat epilepsy, can elevate PUFA levels in epilepsy patients (Auvin, 2012; Fraser et al., 2003). However, it is worth noting that direct PUFA supplementation has not proven effective in epilepsy treatment (Sarmento Vasconcelos et al., 2016). These findings align with our results, which did not indicate a potential causal relationship in the MR analyses of PUFA in focal epilepsy.

Oxidative stress is not only a consequence of seizures but also a key factor in causing them (Fabisiak & Patel, 2022). In the reverse direction, we found that Kyn levels are significant factors predisposing to decreased risk of focal epilepsy. Kyn is a metabolite in the tryptophan metabolic pathway with proconvulsant effects (Heyes et al., 1994). When inflammation produces an increase in cytokines, it activates the regulatory enzyme indoleamine‐2,3‐dioxygenase and related enzymes of the pathway, which leads to dysregulation of the Kyn pathway, allowing the neurotoxic metabolites (quinolinic acid, Kyn, and 3‐hydroxy Kyn) to be elevated, and the neuroprotective agent kynurenic acid decreases (Savitz, 2020). Our findings corroborate a previous study in which elevated cerebrospinal fluid Kyn levels were observed in the epilepsy group in comparison to a control group comprising individuals with neurodevelopmental disorders, psychiatric conditions, and functional neurological disorders (Bai et al., 2022). To the best of our knowledge, our study is the first to intentionally investigate the potential causal connection between Tb levels and epilepsy, particularly in the context of focal NHS. Previous research has primarily concentrated on the impact of anti‐seizure medications, utilizing Tb as an indirect indicator to monitor liver function and refine treatment regimens (Fang et al., 2023; [41,42). Exploring the direct relationship between Tb and epilepsy itself could be a promising avenue for future investigations.

Our study is the first two‐sample MR investigation into the causal relationship between epilepsy and oxidative stress. We obtained the largest sample size of epilepsy GWAS data from the Epilepsy Genetic Association Database of the International League Against Epilepsy for reliable genetic variants (International League Against Epilepsy Consortium on Complex Epilepsies, 2018). We collected biomarkers of oxidative stress injury associated with epilepsy, including Kyn, glutathione, and Ve from the literature (Azam et al., 2012; Geronzi et al., 2018; Pearson‐Smith & Patel, 2017). Furthermore, we combed through all oxidative stress pathways relevant to neuropsychiatric disorders to obtain an overall picture of the interactions between epilepsy and oxidative stress and hopefully gain new insights (Bai et al., 2022; Esmaeili et al., 2022; Lu et al., 2022; Tang et al., 2023).

Our use of MR analysis helps reduce unmeasured confounding and reverse causality, improving the reliability of our findings regarding bidirectional associations. We have also applied a range of MR methods to enhance the rigor and validity of our results, making our study clinically relevant. Nonetheless, it is important to consider certain limitations when interpreting the results. First, the results of this study are based on data from individuals of European ancestry, and whether the results are transferable to non‐European ethnicities needs to be further investigated. Second, the number of SNPs currently identified as instrumental variables for epilepsy and antioxidants is limited, but their ideal representatives have been widely used in previous MR studies (Fang et al., 2023; Lu et al., 2022). Also limited by the number of SNPs for instrumental variables, it was not possible to perform MR‐Egger, weighted‐median, and MR‐PRESSO methods for Tb, PUFA, and so on. Lastly, the potential causality indicated in our study should be approached with caution, given that the *p*‐values are nearly nominal. The absence of an association should also be interpreted judiciously, taking into account the limitations of MR

## 5 CONCLUSION

In summary, the present study demonstrated a bidirectional causal link between epilepsy and oxidative stress from a genetic perspective using MR analysis. This is a crucial realization and highlights that further studies should also consider revealing more precise mechanisms of this relationship. Moreover, this study suggests that in the future, the development of combination therapies that address neuronal hyperexcitability and oxidative stress could prevent the deleterious cycle of neuroinflammation and oxidative stress that leads to epileptogenesis.

## AUTHOR CONTRIBUTIONS

**Yilin Xia**: Conceptualization; methodology; data curation; writing—original draft; writing—review and editing; visualization. **Wanlin Lai and Yifei Duan**: Investigation; data curation; writing—original draft. **Leihao Sha**: Investigation; data curation. **Lei Chen**: Writing—original draft; writing—review and editing; conceptualization; methodology; supervision.

## CONFLICT OF INTEREST STATEMENT

The authors declare no conflicts of interest.

### PEER REVIEW

The peer review history for this article is available at https://publons.com/publon/10.1002/brb3.3549.

## Supporting information

Table S1 GWAS dataset used in the Mendelian randomization.
Table S2 Exposure instruments proxy epilepsy phenotype.
Table S3 Exposure instruments proxy oxidative stress status.
Table S4 MR results of oxidative stress on risk of epilepsy using Wald ratio or inverse‐variance weighted, MR‐Egger, simple mode, weighted‐median, and weighted‐mode.
Table S5 MR results of epilepsy on risk of oxidative stress using Wald ratio or inverse‐variance weighted, MR‐Egger, simple mode, weighted‐median, and weighted‐mode.
Table S6 Pleiotropy analysis of MR of epilepsy on risk of oxidative stress.
Table S7 Heterogeneity analysis of MR of epilepsy on risk of oxidative stress.
Table S8 Leave‐one‐out analysis of MR of epilepsy on risk of oxidative stress.
Table S9 Pleiotropy analysis of MR of oxidative stress on risk of epilepsy.
Table S10 Heterogeneity analysis of MR of oxidative stress on risk of epilepsy.
Table S11 Leave‐one‐out analysis of MR of oxidative stress on risk of epilepsy.
Table S12 Distortion test of MRPRESSO (epilepsy on risk of oxidative stress).
Table S13 Distortion test of MRPRESSO (epilepsy on risk of oxidative stress).

## Data Availability

The data that support the findings of this study are available in Ieu OpenGwas Project at https://gwas.mrcieu.ac.uk/. These data were derived from the following resources available in the public domain: Ieu OpenGwas Project at https://gwas.mrcieu.ac.uk/.
